# Direct Vpr-Vpr Interaction in Cells monitored by two Photon Fluorescence Correlation Spectroscopy and Fluorescence Lifetime Imaging

**DOI:** 10.1186/1742-4690-5-87

**Published:** 2008-09-22

**Authors:** Joëlle V Fritz, Pascal Didier, Jean-Pierre Clamme, Emmanuel Schaub, Delphine Muriaux, Charlotte Cabanne, Nelly Morellet, Serge Bouaziz, Jean-Luc Darlix, Yves Mély, Hugues de Rocquigny

**Affiliations:** 1 Département de Pharmacologie et Physico-Chimie des Interactions Cellulaires et Moléculaires, UMR 7175 CNRS, Faculté de Pharmacie, Université Louis Pasteur, Strasbourg 1, 74, Route du Rhin, 67401 Illkirch Cedex, France; 2 Department of Immunology, The Scripps Research Institute, 10550 North Torrey Pines Road, La Jolla, CA 92037, USA; 3 LaboRétro Unité de Virologie Humaine INSERM 758, IFR 128 Ecole Normale Supérieure de Lyon, 46 allée d'Italie, 69364 Lyon, France; 4 Ecole Supérieure de Technologie des Biomolécules de Bordeaux, Université V Ségalen, Bordeaux 2, 146, rue Léo Saignat, 33076 Bordeaux Cedex, France; 5 Unité de Pharmacologie Chimique et Génétique, Inserm U640 CNRS UMR8151 UFR des Sciences Pharmaceutiques et Biologiques 4, Avenue de L'observatoire, 75006 Paris, France

## Abstract

**Background:**

The human immunodeficiency virus type 1 (HIV-1) encodes several regulatory proteins, notably Vpr which influences the survival of the infected cells by causing a G2/M arrest and apoptosis. Such an important role of Vpr in HIV-1 disease progression has fuelled a large number of studies, from its 3D structure to the characterization of specific cellular partners. However, no direct imaging and quantification of Vpr-Vpr interaction in living cells has yet been reported. To address this issue, eGFP- and mCherry proteins were tagged by Vpr, expressed in HeLa cells and their interaction was studied by two photon fluorescence lifetime imaging microscopy and fluorescence correlation spectroscopy.

**Results:**

Results show that Vpr forms homo-oligomers at or close to the nuclear envelope. Moreover, Vpr dimers and trimers were found in the cytoplasm and in the nucleus. Point mutations in the three α helices of Vpr drastically impaired Vpr oligomerization and localization at the nuclear envelope while point mutations outside the helical regions had no effect. Theoretical structures of Vpr mutants reveal that mutations within the α-helices could perturb the leucine zipper like motifs. The ΔQ44 mutation has the most drastic effect since it likely disrupts the second helix. Finally, all Vpr point mutants caused cell apoptosis suggesting that Vpr-mediated apoptosis functions independently from Vpr oligomerization.

**Conclusion:**

We report that Vpr oligomerization in HeLa cells relies on the hydrophobic core formed by the three α helices. This oligomerization is required for Vpr localization at the nuclear envelope but not for Vpr-mediated apoptosis.

## Background

As for any replication competent retrovirus, the human immunodeficiency virus type 1 (HIV-1) encodes the precursors to the major structural proteins, enzymes and envelope glycoproteins of the viral particle. In addition, HIV-1 codes for essential regulatory factors, notably Tat, Rev and Vpr. Over the past decade, Vpr has been the subject of many studies because it was suspected to play a direct role in the physiopathology of the viral infection. In fact, Vpr was found to interact with the C-terminus of Gag, causing its virion incorporation [1-4], and with cellular proteins in infected cells. Due to these interactions Vpr promotes the transactivation of HIV-1 long terminal repeat (LTR) and can cause a G2/M arrest and apoptosis of cells, but the relationship between these two roles of Vpr is still a matter of debate (reviewed in [5-7]). Also Vpr appears to contribute to the nuclear import of the pre-integration complex (PIC) and thus of the viral DNA [8,9]. This last function is supported by the nuclear envelope (NE) localization of Vpr, which is mediated by interaction with components of the nuclear pore complex (NPC) [10-12].

Vpr is a 96 amino acid protein with an N- terminal domain required for virion incorporation, nuclear localization and oligomerization [13,14]. Its C-terminal domain is involved in the G2/M cell cycle arrest [15], apoptosis [16] and interaction with the viral nucleocapsid protein and nucleic acids [17,18]. Moreover, Vpr-Vpr interaction was shown to be required for nuclear localization but not for cell cycle blockade [19].

**Figure 1:**
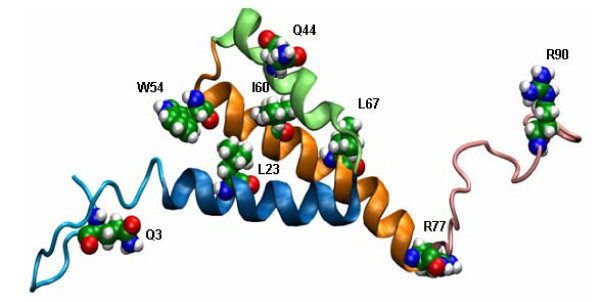
**NMR based structure of Vpr**. The NMR-based 3D- structure of Vpr (1–96) is characterised by three α helices in close vicinity surrounded by flexible N and C termini [22]. Helices are presented in dark blue (17–33), green (38–50) and orange (54–77). Mutated amino acids Q3R, L23A, ΔQ44, W54G, I60A, L67A, R77Q and R90K are represented in CPK mode. Noticeably, the NMR studies were carried out on the Vpr sequence of the HIV-1 pNL43 strain with a Leucine at the position 60 instead of an Isoleucine for the HIV-1_LAI _strain used here. Nevertheless, a predictive study on I60 Vpr showed that the third α helix was not altered compared to L60 Vpr (data not shown).

The 3D structure of Vpr peptides and of full length Vpr in hydrophobic solvents or in the presence of micelles was solved by NMR [20,21]. As illustrated in Figure 1, Vpr is composed of three amphipathic α helices spanning residues (17–33), (38–50) and (54–77), surrounded by flexible N- and C-terminal sequences [22]. Two loops spanning residues (34–37) and (51–53) allow a mutual orientation of these helices, conferring a globular conformation to the protein and promoting the formation of a hydrophobic core with numerous hydrophobic amino acids scattered throughout Vpr. The difficulties encountered to solve the Vpr 3D structure might be explained by its ability to oligomerize via the formation of leucine zipper like motifs [14,23-26].

To further characterize the formation of Vpr oligomers and their intracellular localization, we used eGFP and mCherry Vpr fusion proteins and studied their interaction by two photon fluorescence lifetime imaging microscopy (FLIM) and fluorescence correlation spectroscopy (FCS). We found that Vpr oligomerization relies on both the N- and the C- termini and occurs at the nuclear envelope, in the cytoplasm and in the nucleus. Mutations in the three α helices elicited a large decrease in Vpr-Vpr interaction while mutations in the loops or in the N- or C-termini had little influence on its oligomerization. This study also shows that Vpr oligomerization determines its subcellular localization but not its proapoptotic activity. Finally, molecular modeling of Vpr mutants has been performed in an attempt to draw a possible correlation between Vpr structure and activity.

## Results

### Confocal microscopy visualisation of eGFP or mCherry fused to Vpr N and C termini

In order to monitor Vpr-Vpr interaction by FRET, eGFP or mCherry proteins were fused to Vpr at their C- or N- termini. The eGFP and mCherry were used as a donor/acceptor pair for FRET for several reasons. Firstly, eGFP exhibits a high quantum yield (0.8) and its time resolved fluorescence is characterized by a mono-exponential decay (2.5 ns) [27]. This single exponential decay strongly contrasts with the complex decay of CFP [28], another fluorescent protein commonly used as a donor for FRET, which makes eGFP highly suitable for monitoring FRET due to the decrease of its fluorescence lifetime. Secondly, mCherry was used as the acceptor since its absorption spectrum overlaps the fluorescence spectrum of eGFP, giving a large Förster R_0 _distance (where the transfer efficiency is 50%) of about 54 Å [29]. Moreover, in contrast to the commonly used DsRed protein, mCherry is monomeric and readily matures, which avoids the generation of several proteins with different lifetimes [30]. Lastly, its spectroscopic properties are preserved in mCherry-tagged proteins [31] and its use in association with eGFP to monitor protein/protein interaction by FRET has been validated [28,29,31].

**Figure 2:**
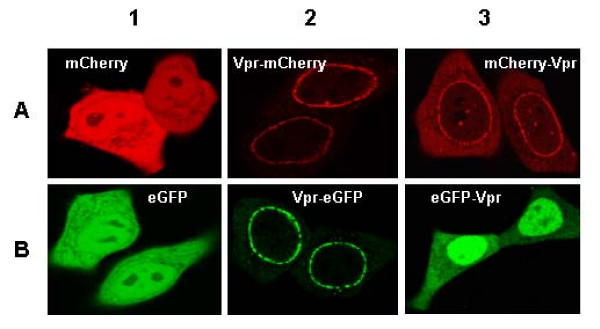
**Subcellular localization of eGFP or mCherry tagged Vpr by confocal microscopy**. HeLa cells were co-transfected with 0.5 μg of each plasmid and 0.5 μg pcDNA3. Cells were observed by confocal microscopy 24 h post transfection. Each panel shows the major phenotype. (A) mCherry images with excitation at 568 nm and emission at 580 to 700 nm. (B) eGFP images with excitation at 488 nm and emission at 500 to 550 nm. Note the intracellular redistribution of eGFP and mCherry upon fusion with Vpr.

Four labelled Vpr proteins were obtained by fusing eGFP or mCherry to Vpr either to its N- or C-terminus. Since both eGFP and mCherry are large with respect to Vpr, we first checked whether the fusion affects the intracellular localization of Vpr. To this end, we analyzed by confocal microscopy at 24 h post transfection the expression of both mCherry- (Figure 2, panels A2-3) and eGFP Vpr fusions in HeLa cells (Figure 2, panels B 2-3). Both Vpr-eGFP and Vpr-mCherry showed a nuclear rim staining coincident with the nuclear envelope (NE) (Figure 2, panels A2 and B2) in agreement with the localization of HA-Vpr (additional file 1, [12]). This localization of Vpr at the NE is not driven by the eGFP and mCherry proteins since both fluorescent proteins were found to be spread all over the cells when expressed in their free form (Figure 2 A1 and B1). Localization of HA-Vpr (additional file 1) or His-Vpr [12] confirms that these proteins are predominantly localized at the nuclear membrane and in the nucleus with some cytoplasmic localization. Thus, the fusion of either mCherry or eGFP to the C terminus of Vpr has a limited effect on Vpr localization in the cell even though the relative proportion of Vpr in the nucleus, at the nuclear envelope or in the cytoplasm was modified [10,12,13,24,32]. The distribution pattern of mCherry-Vpr was close to that of Vpr-mCherry except that a larger amount of protein diffused out in the cytoplasm, indicating a limited alteration of Vpr intracellular distribution by the mCherry fused to the N-terminus of Vpr. In contrast, eGFP-Vpr showed a diffuse distribution in both the cytoplasm and the nucleus (Figure 2, panel B 3) similar to the nuclear staining of eYFP-Vpr [10,12]. At least, it should be mentioned that Vpr distribution was not time dependent since the same pattern of localization was monitored at 48 and 72 h (data not shown).

**Figure 3:**
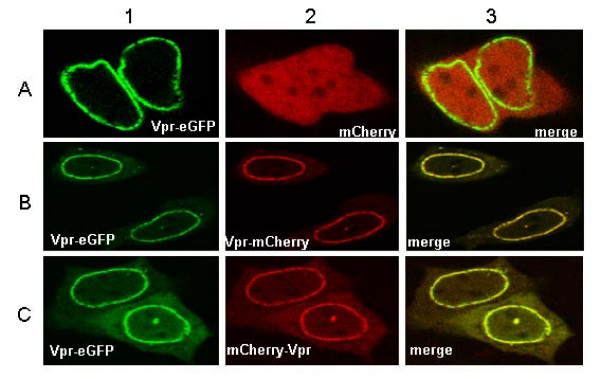
**Visualization of the intracellular co-expression eGFP or mCherry tagged Vpr**. Plasmid DNA (0.5 μg each) expressing the Vpr fusion proteins were cotransfected in HeLa cells. One day post transfection, images were recorded with an excitation at 488 nm and emission at 500–550 nm to monitor eGFP expression, and with an excitation at 568 nm and emission at 580–700 nm to monitor mCherry expression, respectively. In the merge images, co-localization of the two proteins is indicated in yellow. Each image is representative of the major phenotype. Note the accumulation of the Vpr fusion proteins at or close to the nuclear envelope.

Co-localization of Vpr-eGFP and either mCherry-Vpr or Vpr-mCherry was visualized by confocal microscopy. As a control, Vpr-eGFP was first co-expressed with mCherry. Localization of Vpr-eGFP at the nuclear rim (Figure 3, panel A1) was similar to that in Figure 2 (panel B2), indicating that the expression of mCherry did not affect the intracellular distribution of Vpr-eGFP. When Vpr-eGFP was co-expressed with Vpr-mCherry, both green and red fluorescence emissions were localised at the rim of the nucleus and to a lesser extent in the cytoplasm and in the nucleus (Figure 3, panels B1-3). A full co-localization of the two Vpr fusion proteins in the same cellular compartments was further evidenced by the yellow color in Figure 3 (panel B3), that shows a nice superposition of the green and red emissions of the two Vpr fusion proteins. Interestingly, expression of Vpr-eGFP with mCherry-Vpr resulted in a partial redistribution of Vpr-eGFP from the nuclear rim toward the cytoplasm (compare Figure 3, panel C1 with Figure 2, panel B2). The overlap of their emissions all over the cell confirmed their similar intracellular distribution (Figure 3, panel C3).

The re-localization of Vpr-eGFP mediated by mCherry-Vpr in a human cell line suggests that the mCherry-Vpr fusion protein interacts with Vpr-eGFP. However, due to the limited resolution of optic microscopic methods (≈ 200 nm), co-localization does not constitute an absolute proof for direct protein interaction. Direct evidence for the interaction between the eGFP and mCherry Vpr fusion proteins and thus Vpr oligomerization, can be provided by FRET between the two proteins as measured by FLIM.

### Investigating intracellular Vpr-Vpr interaction by FLIM

Due to its exquisite dependence on the inter-chromophore distance, FRET between eGFP- and mCherry tagged proteins will occur only if they are less than 10 nm apart [33,34]. This implies that FRET will only be observed when the tagged proteins directly interact with each other [35,36]. In cells, the FRET efficiency can be directly measured by imaging with the FLIM technique the decrease of the fluorescence lifetime of the donor at each pixel or group of pixels. Indeed, in contrast to fluorescence intensities, the fluorescence lifetimes are absolute parameters that do not depend on the instrumentation or the local concentration of the fluorescent molecules. Thus, changes of the fluorescence lifetimes of the donor will provide a direct evidence for a physical interaction between the labelled proteins with high spatial and temporal resolution [37].

**Figure 4:**
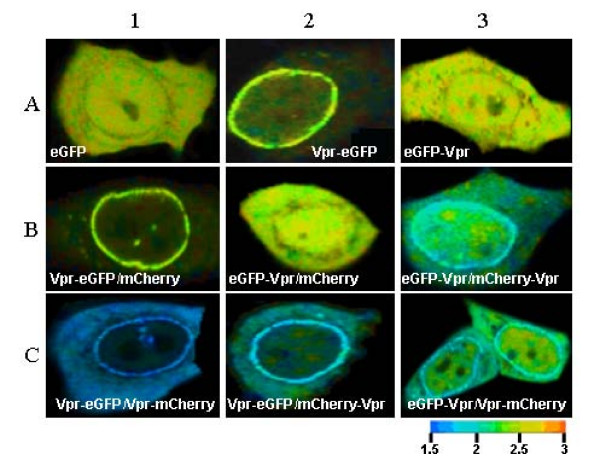
**Direct Vpr-Vpr interaction in HeLa cells visualized by FLIM**. Cells were transfected with the DNA construct encoding eGFP or eGFP-Vpr alone or in combination with mCherry-Vpr. In the FLIM images, the lifetimes are represented using an arbitrary color scale ranging from blue to red for short and long lifetimes in nanoseconds (right bottom), respectively. The Vpr-eGFP or eGFP-Vpr with short lifetime fluorescence symbolized by the blue color were mainly localized at the nuclear envelope and also in other cell compartments when co transfected with mCherry tagged Vpr. Panels A1 to A3 show the lifetime images of cells expressing eGFP or eGFP-tagged Vpr alone. Panels B1 and B2 represent cells coexpressing eGFP-tagged Vpr and mCherry; Panels B3 and C1-C3 show the lifetime images of cells coexpressing eGFP-tagged Vpr and mCherry-tagged Vpr. Note the accumulation of Vpr fusion proteins at or near the nuclear envelope.

HeLa cells were transfected and FLIM measurements were monitored at 24, 48 and 72 hours but since no time dependant effect was monitored; only measurements at 24 h are presented. Experiments were performed first on cells expressing eGFP or Vpr eGFP fusion protein as a control (Figure 4, panels A1-3) and next on cells co-expressing Vpr-eGFP and mCherry fusion proteins (Figure 4, panels B1-3 and C1-3). An arbitrary color scale, ranging from blue to red, illustrates short to long lifetimes. The Vpr-eGFP fluorescence was mainly localized at the nuclear envelope and also in other cell compartments, where FLIM measurements can be carried out. We focused on three distinct regions, namely the nuclear rim, the cytoplasm and the nucleus (Table 1). For the cytoplasm and the nuclear region, care was taken to exclude pixels with contribution from the nuclear envelope. Moreover, due to the thickness of the nuclear envelope, the pixels used to calculate the lifetime values of the nuclear envelope involved contributions from cytoplasmic and nuclear Vpr. Nevertheless, due to the strong accumulation of Vpr at the nuclear membrane, we assumed that the lifetimes mainly reflected the behaviour of the Vpr fusion proteins at this site (see Table 1). FLIM measurements were carried out

**Table 1:** Lifetime and FRET efficiency of eGFP- and eGFP-tagged Vpr in living cells

	**Nuclear envelope**		**Cytoplasm**		**Nucleus**		**Whole Cell**	
	
	E(%)	τ(ns)	E(%)	τ(ns)	E(%)	τ(ns)	E(%)	τ(ns)
eGFP	-	-	-	2.50 (± 0.01)	-	2.50 (± 0.01)	-	2.50 (± 0.01)
Vpr-eGFP	-	2.36 (± 0.01)	-	2.40 (± 0.01)	-	2.41 (± 0.01)	-	2.39 (± 0.01)
eGFP-Vpr	-	2.47 (± 0.01)	-	2.46 (± 0.01)	-	2.47 (± 0.01)	-	2.47 (± 0.01)
Vpr-eGFP+mCherry	-	2.41 (± 0.02)	-	2.42 (± 0.01)	-	2.42 (± 0.01)	-	2.42 (± 0.01)
Vpr-eGFP+Vpr-mCherry	27	1.72 (± 0.02)	23	1.86 (± 0.03)	19	1.95 (± 0.03)	23	1.85 (± 0.03)
Vpr-eGFP+mCherry-Vpr	17	1.95 (± 0.02)	14	2.06 (± 0.02)	13	2.09 (± 0.02)	15	2.02 (± 0.03)
eGFP-Vpr+mCherry	-	2.43 (± 0.01)	-	2.43 (± 0.01)	-	2.43 (± 0.02)	-	2.43 (± 0.01)
eGFP-Vpr+Vpr_mCherry	13	2.14 (± 0.03)	9	2.25 (± 0.03)	6	2.32 (± 0.02)	9	2.25 (± 0.03)
eGFP-Vpr+mCherry-Vpr	13	2.14 (± 0.03)	7	2.28 (± 0.03)	6	2.31 (± 0.02)	8	2.28 (± 0.03)

The fluorescence lifetimes (τ) of eGFP alone or linked to the Vpr C-terminus are the average values (+/- standard deviation) for 10 to 35 cells. For each cell, measurements were performed at the nuclear envelope, in the nucleus and in the cytoplasm. The FRET efficiency (E) is related to the distance between the two chromophores and is calculated from the lifetime ratio with and without the acceptor using equation (2). The whole cell E and τ values represent the average values calculated over the entire cell.

The lifetimes (2.4–2.5 ns) of Vpr eGFP fusion proteins expressed alone (Figure 4, panels A2 and A3) or co-expressed with mCherry (Figure 4, panels B1 and B2) were identical to that of eGFP alone (Figure 4, panel A1) [27]. These results show that the eGFP fluorescence was not altered when fused to Vpr and that no short range interaction occurred between the Vpr eGFP fusion protein and free mCherry.

In contrast, a strong decrease in the average fluorescence lifetime of Vpr-eGFP was observed all over the cell when it was co-expressed with Vpr-mCherry (Figure 4, panel C1), thus indicating a direct physical interaction between the two Vpr chimeric proteins. The strongest decrease was observed at the nuclear rim where the fluorescence lifetime dropped down to 1.72 ns, corresponding to a transfer efficiency of 27% (Table 1). Vpr-Vpr interaction also occurred in the cytoplasm and the nucleus, as shown by the 19–23% energy transfer measured at these sites.

As reported in Table 1 and Figure 4, the energy transfer efficiency is dependent upon the couple of the Vpr fusion proteins. Indeed, the transfer efficiency dropped by a factor of about 1.5 when Vpr-eGFP was co-expressed with mCherry-Vpr (15%; Figure 4, panel C2) and by a factor of about 2.5 when eGFP-Vpr was co-expressed with either Vpr-mCherry (9%; Figure 4, panel C3) or mCherry-Vpr (8%; Figure 4, panel B3). Although Vpr-Vpr interaction was clearly taking place in all cases, a comparison of the energy transfer values suggests that fusion of a fluorescent protein at the Vpr N-terminus is detrimental to Vpr-Vpr interaction.

Taken together, these data indicate that Vpr-Vpr interactions occur in the cytoplasm, in the nucleus and at the nuclear rim and are best visualized when the fluorescent proteins are linked to the C-terminus of Vpr.

### Mapping Vpr-Vpr interaction

In an attempt to map the Vpr domains involved in Vpr-Vpr interaction, site directed mutagenesis was carried out on Vpr-eGFP and Vpr-mCherry constructs based on structural criteria [22] (Figure 1). Several amino acids (L23, Q44, I60 and L67) located in the three α-helices were changed to F (L23F) or A (I60A, L67A) or deleted (ΔQ44). Residues I60 and L67 are involved in Vpr dimerisation through a leucine zipper type motif [21,26]. The L23F and ΔQ44 Vpr mutants retained their ability to translocate to the nucleus but were poorly incorporated into virions [13,24,38].

In parallel, amino acids Q3, and R90 located in the N- and C-flexible termini and residues W54 and R77 located at the extremities of the third helix, were changed to R, K, G, Q respectively (Figure. 1). The Q3R and R77Q mutants were shown to be impaired in their proapoptotic activity and to be associated with long-term non-progressive HIV-1 infection [39,40] while the R90K mutant failed to cause the G2/M cell arrest [41]. Moreover, the W54G mutant was shown to be critical for the interaction with cellular UNG (Uracil DNA glycosilase) and its virion incorporation [41].

**Figure 5:**
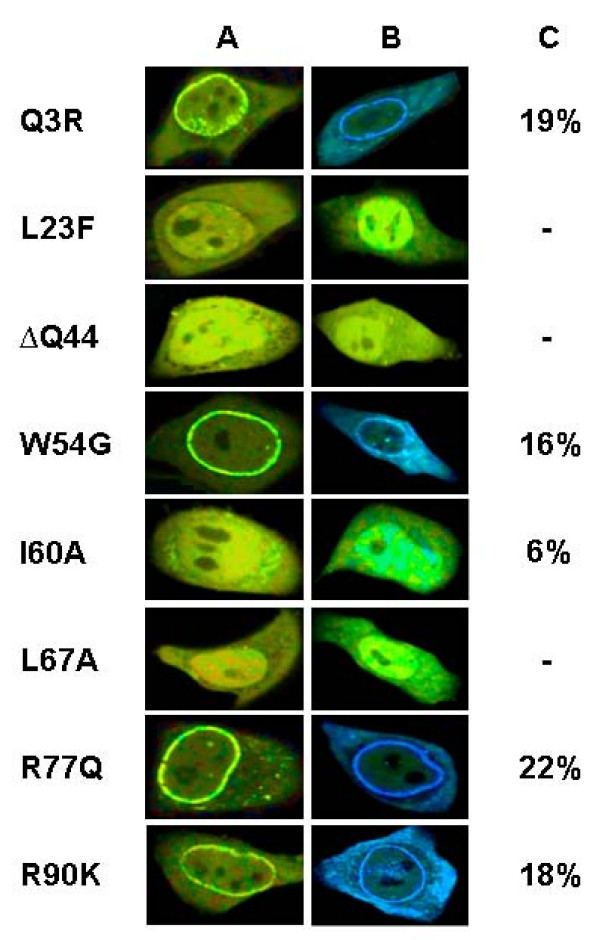
**Mapping of Vpr-Vpr interaction by FLIM**. HeLa cells were co transfected with mutated Vpr-eGFP and its own counterpart fused to mCherry. FLIM was carried out 24 h posttransfection (see methods). Column A corresponds to the FLIM images of the Vpr-eGFP mutants alone, column B to the FLIM images of cells co expressing the mutant Vpr-eGFP and the mutant Vpr-mCherry. FRET efficiency (E) expressed in percentage represents the average value calculated over the entire cell (column C). The color scale used to create theses images is the same than the one used for figure 4. Note the drastic reduction of Vpr-Vpr interaction and the loss of Vpr nuclear envelope accumulation upon mutating residues L23, Q44, I60 and L67 (column B and C).

Mutated proteins were expressed in HeLa cells. Immunodetection by Western Blots revealed that none of the point mutations impeded expression of the Vpr fusion proteins (data not shown). The fluorescence lifetime images were recorded and compared with those of the two wild type Vpr fusion proteins. Figure 5 shows the lifetime images of the Vpr-eGFP mutants expressed in the absence (Column A) and in the presence of the corresponding Vpr-mCherry mutant (Column B). The mean values obtained for the entire cell are reported on the right of the figure. Among the eight mutants, four of them, namely Q3R, W54G, R77Q and R90K, showed a staining pattern similar to that of the wild type fusion proteins with an accumulation at the nuclear rim (compare with Figure 4, panel A2). Oligomers of these mutant proteins were found in the cytoplasm, the nucleus and at the nuclear envelope. The transfer efficiency in the whole cell for these mutants was respectively 19%, 16%, 22% and 18%, similar to the value obtained for the wild type fusion protein (23%). Thus, the Q3, W54, R77 and R90 residues located outside the α-helices are probably not critical for the intracellular localization and oligomerization of Vpr.

On the contrary, the Vpr L23F, ΔQ44, I60A and L67A mutants have lost their ability to accumulate at the nuclear rim. Their intracellular distribution resembled that of eGFP-Vpr, which was evenly distributed in the cell with some accumulation in the nucleus. Interestingly, this different staining pattern of L23F-Vpr-eGFP and ΔQ44-Vpr-eGFP compared to the wild type was also found with L23F-Vpr and ΔQ44-Vpr using immunostaining methodology, indicating that eGFP does not interfere with Vpr distribution [13,24].

A very low transfer efficiency was found for L23F, ΔQ44 and L67A, indicating that these Vpr mutants failed to oligomerize even at or near the nuclear envelope. Thus, the three residues located respectively in the first, second and third helix seemed to be directly involved in Vpr-Vpr interaction and its cellular localization. Furthermore, a small but significant FRET was observed between I60A-Vpr-eGFP and its red counterpart (6% in the whole cell; 7% inside the nucleus- figure 5C) even though the I60A-Vpr-eGFP mutant lost its ability to accumulate at the nuclear envelope. Thus, a minor population of Vpr-eGFP/Vpr-mCherry complex was still observed despite this mutation. In line with this result, transfection of I60A-Vpr-eGFP with wild type Vpr-mCherry restored up to 100% of the nuclear rim staining of the I60AVpr-eGFP mutant (data not shown). Such an important nuclear envelope localization rescue was not observed with the L23F, ΔQ44 and L67A Vpr-eGFP mutants.

Thus, the mapping of Vpr-Vpr interaction reveals that amino acids located in the hydrophobic central core are directly involved in Vpr oligomerization while residues in non-structured domains are dispensable. These results also indicate that the localization of Vpr at the rim of the nucleus probably relies on Vpr-Vpr interaction.

### Vpr oligomerization monitored by FCS

To further characterize Vpr-Vpr interaction in cells, Fluorescence Correlation Spectroscopy (FCS) was performed. This technique characterizes the translational dynamics of fluorescent molecules (or molecular complexes) in any liquid environment. By using the intensity fluctuations of fluorescent species within a femtoliter volume (defined by the laser excitation), several physical parameters – diffusion time, local concentration, molecular brightness, related to the hydrodynamic and photophysical properties of these species – can be monitored [42].

**Figure 6:**
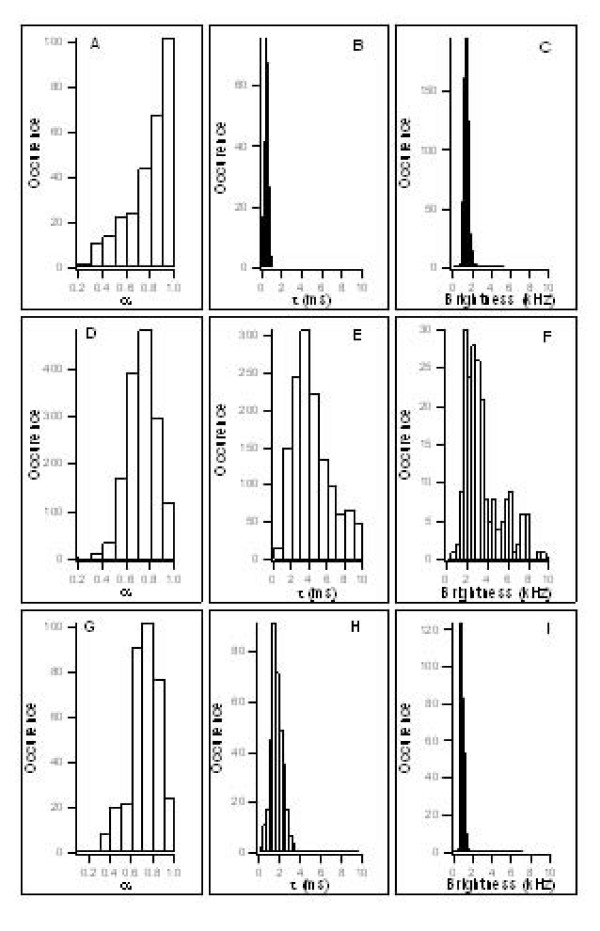
**Distribution histograms of anomalous diffusion coefficients, diffusion times and count rates/species of eGFP, Vpr-eGFP and ΔQ44 Vpr-eGFP**. The anomalous diffusion coefficient (coefficient that accounts for the obstacles encountered by the diffusing species), diffusion times (average time needed to cross the focal volume) and brightness (count rates/species) determined by FCS are expressed as a function of the number of occurrences. A-C correspond to eGFP; D-F correspond to Vpr-eGFP; G-I correspond to ΔQ44 Vpr-eGFP.

Due to the strong eGFP photobleaching, no FCS measurement was possible at the nuclear rim. FCS measurements were thus carried out in the cytoplasm and in the nucleus. Figure 6 reports the histograms of τ_A _(diffusion time), α (anomalous diffusion coefficient) and the count rate per molecule τ_A _represents the average time needed to cross the focal volume, which depends on the size of the molecule or the molecular complex. The α value corresponds to the anomalous diffusion coefficient that accounts for the concentration, size, mobility and reactivity of the obstacles encountered by the diffusing species. Anomalous diffusion was preferred over the two-component diffusion since it takes into account the molecular crowding in the intracellular environment [43]. Moreover, the FCS parameters were obtained from sequential short-time measurements at numerous cell locations to avoid problems due to the non steady-state conditions in cells [42].

Using this protocol, the anomalous diffusion time of eGFP (Figure 6B) displays a narrow distribution centred around 0.4 ms [42], compared to 0.2 ms for purified eGFP in aqueous solution (data not shown). In addition, the α value peaks around 1 (Figure. 6A), suggesting that eGFP freely diffuses as monomers in the cell in agreement with the monomeric structure found by RX [44,45]. A completely different behaviour was observed for Vpr-eGFP. Firstly, the distribution of the apparent diffusion time is shifted to 4 ms (Figure 6E) with dispersion larger than that obtained with eGFP. Since τ_A _roughly varies as the cubic root of the molecular mass of the diffusing species, the tenfold increase of τ_A _implies a thousand fold increased in the molecular mass, unambiguously showing that Vpr fusion proteins form large complexes in cells. Moreover, the anomalous coefficient of Vpr-eGFP presents a distribution centred around 0.75 showing that such complexes do not freely diffuse in the cell but interact with cellular components (Figure 6D). To further characterize these complexes, their molecular brightness (i.e. the number of photons emitted by a particle per second for a given excitation intensity) was compared with that of eGFP (Figure. 6C and 6F). The histogram of eGFP displays a narrow distribution centred around 1 kHz/particle similar to purified eGFP in aqueous solution showing that the photophysical properties of eGFP are not modified by the cellular environment. Since eGFP does not form oligomers, this value can be taken as a reference for eGFP monomers [44,45]. In contrast, the count rate histogram for Vpr-eGFP shows a broad distribution with a major population centred around 2–3 kHz/particle and a minor population with a rather large distribution of brightness (Figure. 6F). This confirms that Vpr forms oligomers as observed by FLIM and suggests that Vpr-eGFP self associates in the cytoplasm and the nucleus notably in the form of dimers and trimers, assuming that the eGFP fluorescence is not modified by Vpr oligomerization. These small oligomers do not explain the aforementioned 10^3^-fold difference between the molar masses of eGFP and Vpr-eGFP complexes, thus indicating that Vpr oligomers probably interact with cellular proteins [46].

FLIM analyses showed that the ΔQ44 mutant of Vpr-eGFP did not interact with Vpr-mCherry (Figure. 5 panel B3). This prompted us to perform FCS experiments with the ΔQ44 Vpr-eGFP to confirm its inability to oligomerize. As shown in Figure 6I, the count rate of ΔQ44 Vpr-eGFP is centred around 1.2 kHz, close to the value obtained for eGFP (Figure. 6C), which confirms that the ΔQ44 Vpr-eGFP does not form oligomers. Interestingly, the diffusion coefficient τ_A _for the Vpr ΔQ44 mutant is about 2 ms (Figure. 6H), a value in between that for eGFP (0.4 ms) and that for Vpr-eGFP (4 ms). Moreover, the distribution of the anomalous coefficient was similar to that for Vpr-eGFP with a peak value around 0.75. The five-fold increase of τ_A _with respect to free eGFP, which corresponds to a 100-fold increase in the molar mass, indicates that this Vpr mutant probably interacts with host proteins in a monomeric form.

### Vpr oligomerization is not necessary for the induction of cell apoptosis

**Figure 7:**
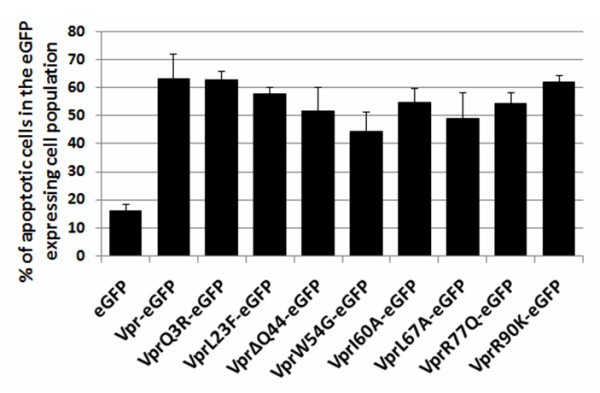
**Pro-apoptotic properties of the Vpr-eGFP mutants**. Cells expressing either the wild type Vpr-eGFP or mutant Vpr-eGFP were selected by fluorescence cytometry, using the eGFP fluorescence. The percentage of cells undergoing apoptosis was assessed by the number of cells labeled with cells with Cy5 alone, or with both Cy5 and PI. Statistical analysis was achieved using the multi-factorial ANOVA test and the Dunnett analysis. Three independent measurements were performed for each assay.

Vpr can induce apoptosis of infected cells and probably of bystander cells [5,6]. In order to evaluate the role of Vpr oligomerization on its pro-apoptotic activity, FACS analyses were carried out. To this end, annexin V and propidium iodide staining of HeLa cells expressing eGFP, Vpr-eGFP or Vpr-eGFP mutants were performed 72 hours after transfection (see methods). Results show that 6% of mock transfected cells (data not shown) and 16% of cells expressing eGFP were apoptotic (Figure. 7). The percentages of apoptotic cells expressing either Vpr-eGFP or one mutant varied from 45 to 70% as compared to the 43% obtained with wt Vpr (data not shown) [12,47]. Thus, no significant reduction of apoptosis was monitored for the Vpr-eGFP mutants examined here. As a consequence there is no clear correlation between the intracellular oligomerization of Vpr and its pro-apoptotic properties.

## Discussion

We report here a study on Vpr oligomerization in the cellular context by confocal microscopy, two photon FCS and FLIM. Using eGFP or mCherry tagged at their N or C terminus by Vpr, we confirmed that Vpr oligomerization occurs in human cells [19], notably at the nuclear envelope (Figure. 3 and 4) in line with the preferential localization of the wild type Vpr [13,24,32,48]. Moreover, FCS experiments also show that Vpr could form two populations of oligomers in the cytoplasm and in the nucleus, one containing mainly dimers and/or trimers and a second composed by a large number of molecules (Figure. 6). This heterogeneity of Vpr oligomers is in agreement with biochemical data showing that the stoichiometry of Vpr oligomers could vary from two to six [14,23,49]. Moreover, FCS analyses of Vpr-eGFP showed that Vpr does not freely diffuse in the cell and thus most probably forms oligomers that interact with cellular proteins [10,11,50-53] and membranes [54,55]. These Vpr oligomers explain the energy transfer observed between eGFP- and mCherry-tagged Vpr proteins by FLIM. The maximum energy transfer was obtained when Vpr was linked to the N terminus of the two reporter proteins (Table 1), which further highlights the role of the N-terminal domain in Vpr oligomerization [14,24].

The 3D structure of Vpr is characterized by three amphipathic α-helices with relative orientations displaying two accessible hydrophobic domains and a hydrophilic one (Figure 1). To map the Vpr-Vpr interactions, we studied Vpr mutants harbouring a single mutation in the helical or flanking regions of the protein [22]. In a first step, we characterized the L23F, I60A and L67A mutations of the hydrophobic central core. Predictive structural studies performed on the mutated protein revealed that these α helices were not significantly altered by these mutations and as a consequence the global 3D structure of the mutant proteins closely resembles that of the wild type Vpr (data not shown).

The L23F and L67A Vpr mutants were distributed throughout the cell, indicating that these residues are critical for addressing Vpr at the nuclear envelope. A similar intracellular distribution of non-fluorescently labelled L23F-Vpr has been previously found by immunofluorescence [13,24], indicating that eGFP did not interfere with the cellular distribution of such Vpr mutants. As the α-helix (17–33) containing the L23 residue is predicted to adopt a coiled-coil conformation, this mutation might well cause an interruption of the leucine stretch formed by residues L20, L22, L23 and L26 located on the same side of the helix (additional file 2). Thus, this hydrophobic platform formed by the N terminal alpha helix (17–33) is a recognition motif for Vpr-Vpr oligomerization in the cellular context.

Mutating residue I60 is less detrimental than mutating residues L23 and L67 for addressing Vpr at the nuclear envelope (Figure 5) since a residual energy transfer was observed. Moreover, the peri-nuclear localization of the I60A-Vpr mutant was recovered upon co-expression with Vpr-mCherry (data not shown). Residues I60 and L67 are involved in a hydrophobic stretch constituted by residues L61, I63, L64, L68, I70 and I74 in the helix (54–77). Since I60 is the first residue of this stretch (additional file 2), changing I to Ala should not affect drastically this hydrophobic motif, and thus Vpr oligomerization and nuclear localization. On the opposite, L67 is located in the center of this hydrophobic motif and changing it to Ala should cause a significant disorder that likely perturbs Vpr oligomerization and nuclear localization. Mutation of residue 67 and the loss of Vpr-Vpr binding was reported and explained by the presence of the negative charge of the glutamic residue placed at this position [19]. Nevertheless, since alanine differs only moderately from leucine it appears that the length and the hydrophobicity of the leucine side chain is critical for maintaining the leucine zipper like structure and the hydrophobic core of Vpr.

**Figure 8:**
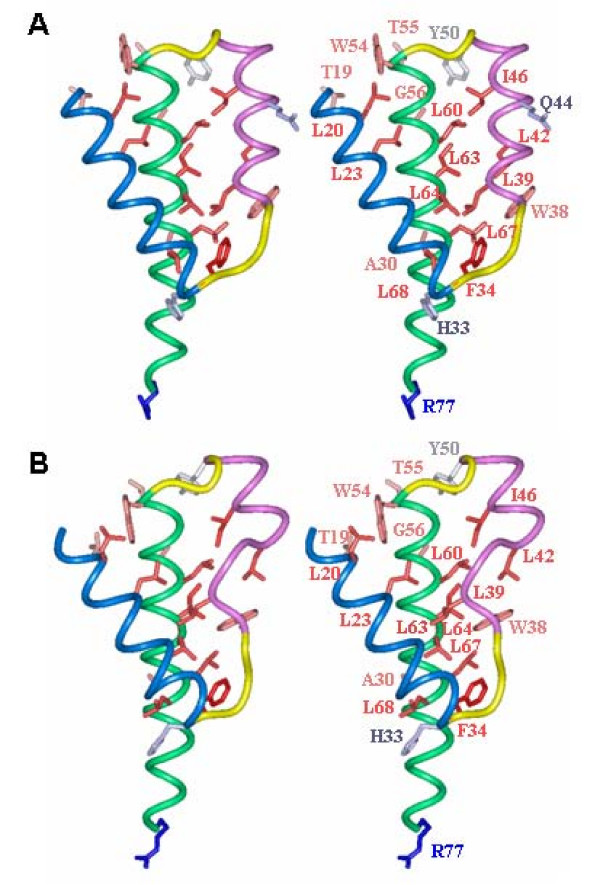
**Comparison of the wild type and ΔQ44 Vpr mutant structures**. Stereoview of the three dimensional structure of the wild type Vpr determined by NMR (A) and theoretical model for the Vpr ΔQ44 mutant (B). Helices (17–33), (38–50) and (54–77) are represented as ribbon and colored in blue, pink and green, respectively and loops (34–37) and (51–53) are colored in yellow. For clarity, the two disordered extremities of the molecule have not been represented. Residues showing long range correlations on NOESY NMR experiments have been displayed in the stick representation and colored according to their hydrophobicity. Only their side chain atoms have been represented. The network of hydrophobic residues can be observed at the interface of the three α-helices. Note the impact of the ΔQ44 deletion (B) on the partial unfolding of the second helix and the rearrangement of the hydrophobic residues at the interface.

The ΔQ44 mutation drastically impaired the oligomerization and localization of Vpr at the nuclear envelope, further suggesting a direct correlation between these two phenomena. Molecular modeling of this mutant shows a partial unfolding of the second helix, from residues W38 to L42 (Figure 8). These structural modifications reorient the residue side-chains involved in the hydrophobic interactions within helix (54–77). Thus, the hydrophobic core formed by hydrophobic stretches of the second and third helices is disrupted and reorganized, leading to a strong modification of the overall Vpr structure. This altered structure might explain why the mutant Vpr has lost its ability to form oligomers and its localization at the nuclear envelope.

In contrast to the aforementioned mutants, a wild type Vpr docking at the nuclear rim was observed for mutations in the loops (W54G, R77Q) and the flexible N- and C- terminal regions (Q3R, R90K), indicating that these residues are dispensable for Vpr cellular localization. In addition, molecular modeling indicates that these mutations should not modify the overall structure of Vpr (data not shown).

Targeting of Vpr at the nuclear envelope most probably relies on its interaction with components of the nuclear pore complex (NPC) [8,11] and especially with the nucleoporin hCG1 [10]. The Vpr/hCG1 interaction is mediated by the hydrophobic core of Vpr independently of its N- and C-termini [10]. For instance, the L23F mutation that alters the Vpr-hCG1 complex was recently shown to cause a lack of Vpr accumulation at the nuclear rim [38]. Thus, the hydrophobic residues of Vpr core are most probably required both for Vpr-Vpr and Vpr-hCG1 interactions. It can thus be speculated that Vpr-hCG1 recognition depends on Vpr oligomerization.

The role of the nuclear localization and oligomerization of Vpr on the induction of apoptosis was studied. In fact, Vpr-eGFP is still able to induce apoptosis, indicating that eGFP does not impair the Vpr apoptotic activity [12]. Similar levels of apoptosis were found for all Vpr mutants. The apoptotic activity of the Q3R and R77Q Vpr mutants are in variance with published reports [39,40] but could be explained by a possible eGFP-mediated gain of function [12,47]. The apoptotic activity of all Vpr mutants shows that this activity is not correlated with Vpr oligomerization. Meanwhile, Bolton and Lenardo have recently showed that Vpr oligomerization of Vpr was dispensable for mediating G2/M arrest [19].

## Conclusion

Taken together, our data show that i) Vpr oligomerizes in the nucleus and the cytoplasm in HeLa cells, ii) Vpr oligomerization is required for Vpr localization at the nuclear envelope, iii) the structural determinants for Vpr oligomerization are located in the hydrophobic core formed by the three α helices and iv) nuclear localization and oligomerization are neither required nor sufficient for apoptosis as for G2/M cell cycle arrest [19].

## Methods

### Plasmid DNA construction

Construction of eGFP-Vpr and Vpr-eGFP was previously described [32]. To construct mCherry-Vpr, we PCR amplified the full length coding sequence of Vpr (from HIV-1_LAI_) using the mammalian HA-tagged Vpr expressing vector [56]. The reverse primer ^5'^GCCCCGCTCGAGCT AGGATCTACTGGC^3' ^used in the PCR amplification was designed to include an *XhoI *restriction site (underlined). The PCR DNA product was digested by *EcoRV *and *XhoI *and cloned into a mCherry expression vector under the control of the CMV promoter.

The Vpr-mCherry recombinant was constructed by a two step protocol. Firstly, the full length coding sequence of Vpr was amplified by PCR from the HA-tagged Vpr expression vector described above. The forward ^5'^CCCAAGCTT**GATCTACC**ATGGAACAAGCCCCAGAAG^3' ^and reverse ^5'^CGCGGATCCCCGGATCTACTGGCTCCATTTC^3' ^primers were designed to include the restriction sites *HindIII *and *BamHI *(underlined). The complementary sequence corresponding to the Kozak consensus for optimal translation initiation is shown in bold. The PCR fragment was digested and cloned into pDsRED-Monomer-N1 (Clontech) to obtain Vpr-DsRED. Secondly, the DsRED coding sequence was cut out with *BamHI *and *NotI *and replaced by the mCherry coding sequence. The latter was amplified by PCR from a mCherry expressing plasmid using the following primers: ^5'^CGCGGATCCAGGAGGCGGTGGGATGGTGAGCAAGGGCGAG^3' ^and ^5'^ATA GTTTAGCGGCCGCTTACTTGTACAGCTCG TCCATGCC^3'^.

Deletion or substitution mutants were carried out by PCR based site-directed mutagenesis on the Vpr-eGFP and Vpr-mCherry expressing vector using a protocol from Stratagene.

### Cell culture and DNA transfection

HeLa cells (10^5^) were cultured on 35 mm glass coverslips (μ-Dish IBIDI, Biovalley, France) in Dulbecco's modified eagle medium supplemented with 10% fetal calf serum (Invitrogen Corporation, Cergy Pontoise, France) at 37°C in a 5% CO_2 _atmosphere. Transfection of HeLa cells with 0.5 μg of each plasmid was achieved with FuGENE™ 6 transfection agent (Roche) or jetPEI™ (PolyPlus transfection, Illkirch, France) according to supplier's recommendations. To keep a constant amount of transfected DNA, each transfection assay was supplemented with the necessary amount of pcDNA3 (Invitrogen Corporation, Cergy Pontoise, France) up to 1 μg of total DNA.

### Immunodetection of Vpr and Vpr derivatives by Western blotting

HeLa cells (2 × 10^5^), transfected with 3 μg of plasmids expressing either eGFP, Vpr-eGFP or Vpr-eGFP mutants, were treated with trypsin and resuspended in ice cold lysis buffer (1% Triton X-100, 100 mM NaF, 10 mM NaPPi, 1 mM NA_3_VO_4 _in PBS supplemented with a complete anti-protease cocktail from Roche, Meylan, France). After sonication and centrifugation, total protein concentrations were assessed by a Bradford assay (Bio-Rad). 25 μg of total proteins were added into 10 mM DTT containing loading buffer (Laemmli, Bio-Rad), heat denaturated and electrophoresed on 12% SDS-PAGE gel. Subsequently, proteins were transferred onto a polyvinylidene difluoride (PVDF) membrane (Amersham, Orsay, France) and blots were probed with an anti-GFP antibody (Clontech) followed by horseradish peroxidase-conjugated anti-mouse antibody. Visualization of proteins was carried out using the chemiluminescent ECL system (Amersham).

### Flow cytometry

Induction of cell apoptosis by Vpr was monitored using Annexin V and propidium iodide (PI) staining. Briefly, 2 × 10^5 ^HeLa cells were transfected with plasmids encoding either eGFP, Vpr-eGFP or Vpr-eGFP mutants. Seventy-two hours posttransfection, the cells were detached, washed in ice cold PBS and resuspended in binding buffer (10 mM Hepes, 140 mM NaCl, 2.5 mM CaCl_2_, pH 7.4). After addition of 5 μl of Annexin V-Biotin, 10 μl of PI (50 μg/ml) and 0.5 μg of streptavidin-Cy5 diluted in 100 μl of binding buffer, the cells were incubated in the dark for 15 minutes. The volume of each tube was brought up to 500 μl with 1× binding buffer. The cells were analyzed by flow cytometry on a FACS Calibur (Becton Dickinson) within a one hour period. In the eGFP positive cell population, the percentage of apoptotic cells was determined from the number of fluorescently labeled cells with Cy5 alone, or with both Cy5 and PI.

### Confocal Microscopy

Fluorescence confocal images of Vpr tagged proteins in living cells were taken 24, 48 and 72 h posttransfection using a confocal microscope (SPC UV1 AOBS, Leica) equipped with a HCX PL APO CS 63× oil immersion objective and an Ar/Kr laser. The eGFP images were obtained by scanning the cells with a 488 nm laser line and filtering the emission with a 500 to 550 nm band-pass. For the mCherry images, a 568 nm laser line was used in combination with a 580 to 700 nm band-pass filter.

#### Immunofluorescence study

HeLa cells were transfected with 0.5 μg of HA-tagged Vpr expressing vector [56] in Labtek (Nunc, Fisher Scientific Bioblock, France). At 24 h, the cells were washed in PBS at 4°C, fixed with paraformaldehyde/PBS (3.5%, w/v), washed again with PBS and permeabilized with 0.2% triton/PBS. After drying, the cells were blocked for 30 min with BSA-PBS 4% and then incubated with anti HA (1/1000) (Invitrogen Corporation, Cergy Pontoise, France) overnight at 4°C. The cells were washed with PBS and incubated with FITC anti-rabbit at 1/200 (Invitrogen Corporation, Cergy Pontoise, France) for 60 min at room temperature. After washing, cells were analysed by confocal microscopy (Bio-Rad 1024, Kr/Ar laser 488/568).

### Fluorescent Correlation Spectroscopy (FCS)

FCS measurements were performed on a home-build two-photon system set-up based on an Olympus IX70 inverted microscope with an Olympus 60× 1.2NA water immersion objective as previously described [57,58]. Two-photon excitation at 900 nm was provided by a mode-locked titanium-saphire laser (Tsunami, Spectra Physics). The normalized autocorrelation function was calculated on-line with a hardware correlator (ALV5000, ALV GmbH, Germany). Due to the inherent heterogeneity of the cellular medium, the FCS data were interpreted in terms of anomalous diffusion. Therefore curves were fitted according to:

(1) $$\text{G}\left(\tau \right)=\frac{\text{1}}{\text{N}}{\left(\text{1}-{\left(\frac{\tau }{\tau_{\text{A}}}\right)}^{\alpha }\right)}^{-\text{1}}\cdot {\left(\text{1}+{\left(\frac{\tau }{\tau_{\text{A}}}\right)}^{\alpha }\cdot \frac{\text{1}}{{\text{S}}^{\text{2}}}\right)}^{-\text{1/2}}$$

where N is the average number of fluorescent species in the focal volume, τ the lag time, τ_A _the average residence time in the focal volume, α the anomalous diffusion coefficient and S a structural parameter defined as the ratio between the axial and lateral radii of the beam waist. The molecular brightness (η) of the fluorescent species diffusing through the excitation volume is obtained by dividing the average fluorescence intensity <F> by N. In free lateral diffusion (α = 1), the mean-square displacement of the diffusing species is proportional to time (<r^2^>~t). This is no more valid for anomalous diffusion (α < 1), that takes place in systems containing obstacles. In that case, the mean-square displacement is described by a power law (<r^2^>~t^α^) with a coefficient α depending on the concentration, size, mobility and reactivity of the obstacles. Moreover, in living cells, there is no real steady-state for the fluorescence intensity fluctuations. For this reason, FCS measurements were sequentially repeated, typically 40 × 5 s. Each FCS curve is then fitted independently. A Labview program was written to automatically process the data. The results are represented by histograms of the fitting parameters.

### Fluorescence Lifetime Imaging Microscopy (FLIM)

Time-correlated single-photon counting FLIM was performed using an in house constructed multi-photon laser scanning microscope sharing the same core as the system described for FCS measurements. For FLIM, the laser power was adjusted to give count rates with peaks up to as few as 10^6 ^photons.s^-1^, so that the pile-up effect can be neglected. Imaging was carried out with a laser scanning system using two fast galvo mirrors (Model 6210, Cambridge technology), operating in the descanned fluorescence collection mode.

Photons were collected using a set of two filters: a two-photon short pass filter with a cut-off wavelength of 680 nm (F75-680, AHF, Germany), and a band-pass filter of 520 ± 17 nm (F37-520, AHF, Germany). The fluorescence was directed to a fiber coupled APD (SPCM-AQR-14-FC, Perkin Elmer), which was connected to a time-correlated single photon counting (TCSPC) module (SPC830, Becker & Hickl, Germany), which operates in the reversed start-stop mode.

Typically, the samples were scanned continuously for about 30s to achieve appropriate photon statistics to analyse the fluorescence decays. Data were analysed using a commercial software package (SPCImage V2.8, Becker & Hickl, Germany), which uses an iterative reconvolution method to recover the lifetimes from the fluorescence decays.

In Fluorescence Resonant Energy Transfer (FRET) experiments, when co-expressing donor and acceptor proteins, the FRET efficiency reflecting the distance between the two chromophores was calculated according to:

(2) $$\text{E}=\frac{{\text{R}}_{\text{0}}^{\text{6}}}{{\text{R}}_{\text{0}}^{\text{6}}+{\text{R}}^{\text{6}}}=\text{1}-\frac{\tau_{\text{DA}}}{\tau_{\text{D}}}$$

where R_0 _is the Förster radius, R the distance between donor and acceptor, τ_DA _is the lifetime of the donor in the presence of the acceptor, and τ_D _is the lifetime of the donor in the absence of the acceptor.

### Molecular modeling of Vpr mutants

The impacts of the L23F mutation in the first α-helix [17-33], the ΔQ44 deletion in the second helix [38-50] and the I60A and L67A mutations in the third α-helix [54–77] on the 3D structure of Vpr have been investigated by in silico procedure. Calculations were performed on a SGI Octane work station with the Discover/NMRchitect software (Accelrys, Inc. San Diego, CA, USA). Each mutation has been introduced in the wild type Vpr structure and each of the four resulting structures has been submitted to a 500 steps of steepest descent followed by a 5000 steps of conjugate gradient minimization until a maximum gradient value of 0.01 kcal/mol/Å was reached. Calculations were performed on a SGI Octane station with the Discover/NMRchitect software package from Accelrys. Each generated mutant structure was analyzed by comparison with the wild type NMR structure using the InsightII program visualization. No NMR distance or angle restraints were used during minimization.

## Abbreviations

FRET: Fluorescence Resonance Energy Transfer; FCS: Fluorescent Correlation Spectroscopy; FLIM: Fluorescence Lifetime Imaging Microscopy; WB: Western Blot; FACS: Fluorescence-activated cell sorting

## Competing interests

The authors declare that they have no competing interests.

## Authors' contributions

JVF did all the experiments and analysis of the data, PD, JPC and ES set up the platform for FCS and FLIM, CC produced eGFP for in vitro controls, DM gave plasmid and expertise for cellular studies, SB and NM performed molecular modelling, JLD and YM made substantial contribution for data interpretation and manuscript writing and HR designed and monitored the study. All the authors have read and approved the manuscript.

## Supplementary Material

Additional file 1 **Subcellular distribution of HA-Vpr by immunodetection**: HeLa cells were transiently transfected by 0.5 μg of pHA-Vpr. At 24 h postransfection, cells were incubated with a monoclonal anti-HA antibody followed by incubation with a fluorescein labelled anti-rabbit antibody. Representative thin section of the localization patterns observed by confocal microscopy is shown. Click here for file

Additional file 2 **Surface representation of the wild type Vpr structure showing the two putative hydrophobic platforms for Vpr oligomerization**. The two platforms available for Vpr oligomerization, in the first and third helices, have been colored in red and hydrophobic residues represented in the CPK mode. (A) Localization of the hydrophobic residues, L20, L22, L23 and L26, constituting the leucine zipper motif in the first helix. Arrow indicates the residue L23 important for the hydrophobic platform integrity and consequently for Vpr oligomerization. (B) Hydrophobic platform constituted by residues I60, I61, L63, L64, L67, L68, I70 and I74 located in the third helix. Arrows indicate the two residues I60 and L67, located respectively at the edge and in the center of the platform. Mutation of I60 to Alanine has a less drastic effect on Vpr oligomerization compared to the mutation of L67 into Alanine. Click here for file
