# Supplementary figures and images for: Direct Vpr-Vpr Interaction in Cells monitored by two Photon Fluorescence Correlation Spectroscopy and Fluorescence Lifetime Imaging
Source: Retrovirology. 2008 Sep 22;5:87. doi: 10.1186/1742-4690-5-87 (PMC2562391; doi:10.1186/1742-4690-5-87)

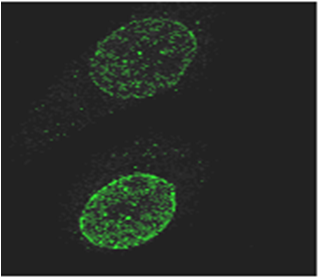

Supplement: Additional file 1 — Subcellular distribution of HA-Vpr by immunodetection: HeLa cells were transiently transfected by 0.5 μg of pHA-Vpr. At 24 h postransfection, cells were incubated with a monoclonal anti-HA antibody followed by incubation with a fluorescein labelled anti-rabbit antibody. Representative thin section of the localization patterns observed by confocal microscopy is shown. [file 1742-4690-5-87-S1.tiff]

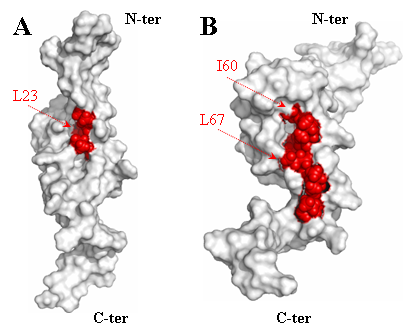

Supplement: Additional file 2 — Surface representation of the wild type Vpr structure showing the two putative hydrophobic platforms for Vpr oligomerization. The two platforms available for Vpr oligomerization, in the first and third helices, have been colored in red and hydrophobic residues represented in the CPK mode. (A) Localization of the hydrophobic residues, L20, L22, L23 and L26, constituting the leucine zipper motif in the first helix. Arrow indicates the residue L23 important for the hydrophobic platform integrity and consequently for Vpr oligomerization. (B) Hydrophobic platform constituted by residues I60, I61, L63, L64, L67, L68, I70 and I74 located in the third helix. Arrows indicate the two residues I60 and L67, located respectively at the edge and in the center of the platform. Mutation of I60 to Alanine has a less drastic effect on Vpr oligomerization compared to the mutation of L67 into Alanine. [file 1742-4690-5-87-S2.tiff]
